# Inversion of Wind-Stress Drag Coefficient in Simulating Storm Surges by Means of Regularization Technique

**DOI:** 10.3390/ijerph16193591

**Published:** 2019-09-25

**Authors:** Junli Xu, Yuhong Zhang, Xianqing Lv, Qiang Liu

**Affiliations:** 1 School of Mathematics and Physics, Qingdao University of Science and Technology, Qingdao 266100, China; xjlshy1983@163.com; 2 College of Engineering, Ocean University of China, Qingdao 266100, China; yuhong3551@126.com; 3 Key Laboratory of Physical Oceanography (Ocean University of China), Ministry of Education, Ocean University of China, Qingdao 266100, China; xqinglv@ouc.edu.cn

**Keywords:** storm surge, adjoint assimilation, regularization technique, typhoon, drag coefficient

## Abstract

In this study, water levels observed at tide stations in the Bohai Sea, Yellow Sea, and East China Sea during Typhoons 7203 and 8509 were assimilated into a numerical assimilation storm surge model combined with regularization technique to study the wind-stress drag coefficient. The Tikhonov regularization technique with different regularization parameters was tested during the assimilation. Using the regularization technique, the storm surge elevations were successfully simulated in the whole sea areas during Typhoons 7203 and 8509. The storm surge elevations calculated with the regularization technique and the elevations calculated with independent point method were separately compared with the observed data. Comparison results demonstrated that the former was closer to the observed data. The regularization technique had the best performance when the regularization parameter was 100. The spatial distribution of the inverted drag coefficient, storm surge elevations, and the wind fields during both typhoons were presented. Simulated results indicated that the change of drag coefficient is more significant in the coastal regions of the Bohai Sea and north of the Yellow Sea. Further analysis showed that the rising water elevation in the Bohai Sea is mostly attributed to the influence of onshore winds, and the negative storm surge in the South Yellow Sea is mainly caused by offshore winds.

## 1. Introduction

Abnormal sea level rise or fall associated with storm surge usually results from strong winds and atmospheric perturbations [1,2,3,4]. The storm surge generated by the typhoon originating from the northwestern Pacific Ocean can result in large scale flooding and destruction on the shore of the Bohai Sea, Yellow Sea, and East China Sea. Storm surge and related coastal flooding can bring about not only damage to properties but also loss of lives [5,6,7,8,9], and the abnormal low water level threatens the maritime safety and coastal facility [10,11,12].

Typhoons can give rise to serious storm surges in the coastal areas. To improve the storm surge forecast, some researchers have studied storm surges in some regions using different methods in recent years. Lionello et al. utilized the variational data assimilation method to forecast the storm surge in the north of Adriatic Sea, showing that the assimilation method can effectively improve the reliability of the storm surge forecast [13]. Peng and Xie combined a linear model with adjoint model of Princeton Ocean Model to present a four-dimensional variational assimilation method [14]. On the basis of this adjoint optimal technique, Peng et al. adjusted the surface boundary and initial conditions in the Princeton Ocean Model, and suggested that it was better to simultaneously adjust those two conditions in the process of data assimilation due to uncertainty of error [15]. Using a coupled model between storm surge and wave, five storm surges from typhoon were simulated in the East China Sea [16]. Results showed that waves should be considered in predicting storm surges. Fan et al. utilized the adjoint assimilation method to optimize the drag coefficient at independent points, and obtained the spatially varying drag coefficient in the Bohai Sea, Yellow Sea, and East China Sea [17]. To improve storm surge forecast, Li et al. optimized the drag coefficient and initial condition using the three-dimensional numerical and adjoint ocean model, and their result demonstrated that simultaneously adjusting both of them can achieve much more improvements [18]. Feng et al. studied the possible influence of future climate changes on storm surges along the Qingdao coast. Results showed that sea level rise mainly influenced the water level during storm surges, while the sea surface temperature affected the intensity of the surges [19]. Xu et al. investigated the impacts of tide-surge interactions on storm surges in the coast of the Bohai Sea, Yellow Sea, and East China Sea using a two-dimensional coupled tide-surge model. It indicated that the tide-surge interactions should be considered when predicting storm surge levels [20].

Nevertheless, the ill-posed problem of solution often appears in the process of adjoint assimilation. In order to solve this problem, regularization technique is introduced [21]. The Tikhonov regularization technique is commonly used, and also proves to be effective in many practical problems [22,23].

For the storm surge forecast, accurate wind data are important in storm surge forecasts. Winds from numerical weather models are often found to be weaker than observations [24]. The variations of wind field to a large extent affect the sea water level. Meanwhile, the drag coefficient is also an important factor in the process of simulating the storm surge. Therefore, in the present study, by assimilating water levels observed at tide stations during Typhoons 7203 and 8509 into a storm surge model, we will optimize the drag coefficient in the Bohai Sea, Yellow Sea, and East China Sea using the Tikhonov regularization technique with different regularization parameters, and examine the spatially varying drag coefficient in these areas. Furthermore, the storm surge elevations simulated with the Tikhonov regularization technique and the elevations calculated with independent point method [17] are separately compared with the observed data. The spatially varying wind stress drag coefficient with wind speed is obtained by the regularization method, thus providing the service for the accurate simulation and prediction of storm surges.

## 2. Materials and Methods

### 2.1. Numerical Adjoint Model

The numerical adjoint model includes two parts: the forward model and adjoint model. The former is a depth averaged two-dimensional storm surge model governed by the depth averaged continuity equation and momentum equations [17].

(1) $$\frac{\partial \zeta }{\partial t}+\frac{\partial \left[\left(h+\zeta \right)u\right]}{\partial x}+\frac{\partial \left[\left(h+\zeta \right)v\right]}{\partial y}=0,$$

(2) $$\frac{\partial u}{\partial t}+u\frac{\partial u}{\partial x}+v\frac{\partial u}{\partial y}-fv+\frac{ku\sqrt{u^{2}+v^{2}}}{h+\zeta }-A\left(\frac{\partial^{2}u}{\partial x^{2}}+\frac{\partial^{2}u}{\partial y^{2}}\right)+g\frac{\partial \zeta }{\partial x}+\frac{1}{\rho_{w}}\frac{\partial P_{a}}{\partial x}-\frac{\rho_{a}}{\rho_{w}}\frac{C_{d}W_{x}\sqrt{W_{x}^{2}+W_{y}^{2}}}{h+\zeta }=0,$$

(3) $$\frac{\partial v}{\partial t}+u\frac{\partial v}{\partial x}+v\frac{\partial v}{\partial y}+fu+\frac{kv\sqrt{u^{2}+v^{2}}}{h+\zeta }-A\left(\frac{\partial^{2}v}{\partial x^{2}}+\frac{\partial^{2}v}{\partial y^{2}}\right)+g\frac{\partial \zeta }{\partial y}+\frac{1}{\rho_{w}}\frac{\partial P_{a}}{\partial y}-\frac{\rho_{a}}{\rho_{w}}\frac{C_{d}W_{y}\sqrt{W_{x}^{2}+W_{y}^{2}}}{h+\zeta }=0,$$

where t is time, x and y are separately the Cartesian coordinates pointing to the east and north, h is unperturbed water depth, ζ is sea surface level, with respective to the unperturbed depth, h+ζ is total depth of water, u and v are separately the current speeds towards the east and north, f=2Ωsinφ is the Coriolis parameter (Ω is earth spinning angular velocity, and φ is north latitude), k is bottom friction factor, A is eddy viscosity coefficient in the horizontal direction, g is gravitational acceleration. Seawater density $\rho_{w}$ is 1025 kg/m^3^, and air density $\rho_{a}$ is 1.27 kg/m^3^. $C_{d}$ is wind-stress drag coefficient, ($W_{x}$,$W_{y}$) is surface wind field and $P_{a}$ is air pressure on the sea surface.

In the present study, the wind field of Jelesnianski [25] was used for the tropical typhoon and is expressed as follows:

(4) $$\vec{W}=\{\begin{array}{c} \frac{r}{R+r}\left(V_{ox}\vec{i}+V_{oy}\vec{j}\right)+W_{R}\frac{1}{r}{\left(\frac{r}{R}\right)}^{\frac{3}{2}}\left(A\vec{i}+B\vec{j}\right),\begin{array}{cc} & 0<r\leq R \end{array}\\ \frac{R}{R+r}\left(V_{ox}\vec{i}+V_{oy}\vec{j}\right)+W_{R}\frac{1}{r}{\left(\frac{R}{r}\right)}^{\frac{1}{2}}\left(A\vec{i}+B\vec{j}\right),\begin{array}{cc} & r>R \end{array} \end{array},$$

where the unit vectors $\vec{i}$ and $\vec{j}$ point to the east and north, respectively, $V_{ox}$ and $V_{oy}$ are the travelling velocities of storm center, r is the distance of the grid center from the storm center, R is radius of the maximum wind speed $W_{R}$

(5) $$A=-\left[\left(x-x_{c}\right)\sin\theta +\left(y-y_{c}\right)\cos\theta \right],$$

(6) $$B=\left[\left(x-x_{c}\right)\cos\theta -\left(y-y_{c}\right)\sin\theta \right],$$

where the coordinate (x,y) is the grid center, $\left(x_{c},y_{c}\right)$ is the storm center; θ is the inflow angle, as the following

(7) $$\theta =\{\begin{array}{c} 20^{\circ },\begin{array}{cc} & r\leq R \end{array}\\ 15^{\circ },\begin{array}{cc} & r>R \end{array} \end{array}.$$

The pressure field of tropical cyclone is originated from Jelesnianski [25]:

(8) $$P_{a}=\{\begin{array}{c} P_{0}+\frac{1}{4}\left(P_{\infty }-P_{0}\right){\left(\frac{r}{R}\right)}^{3},\begin{array}{cc} & r\leq R \end{array}\\ P_{\infty }-\frac{3}{4}\left(P_{\infty }-P_{0}\right)\left(\frac{R}{r}\right),\begin{array}{cc} & r>R \end{array} \end{array},$$

where the pressure $P_{a}$ is at r on the sea surface, pressure $P_{0}$ is at the cyclone center, $P_{\infty }$ is ambient pressure. Here $P_{\infty }$ fetches the value of 1020 hPa.

To build the adjoint equations, the cost function uses the following definition:

(9) $$J\left(\zeta \right)=\frac{1}{2}K_{\zeta }\int_{\Sigma }{\left(\zeta -\hat{\zeta }\right)}^{2}dxdydt,$$

where ζ is the simulation, $\hat{\zeta }$ is the observation, $K_{\zeta }$ is a constant. Then, the Lagrangian function is defined as follows:

(10) $$L=J\left(\zeta \right)+\int_{\Sigma }\zeta_{a}\left\{\frac{\partial \zeta }{\partial t}+\frac{\partial \left[\left(h+\zeta \right)u\right]}{\partial x}+\frac{\partial \left[\left(h+\zeta \right)v\right]}{\partial y}\right\}dxdydt\;+\int_{\Sigma }u_{a}\left[\frac{ku\sqrt{u^{2}+v^{2}}}{h+\zeta }-A\left(\frac{\partial^{2}u}{\partial x^{2}}+\frac{\partial^{2}u}{\partial y^{2}}\right)+\frac{1}{\rho }\frac{\partial p_{a}}{\partial x}-\frac{\rho_{a}}{\rho }\frac{C_{d}W_{x}\sqrt{W_{x}{}^{2}+W_{y}{}^{2}}}{h+\zeta }\right]dxdydt\;+\int_{\Sigma }v_{a}\left[\frac{kv\sqrt{u^{2}+v^{2}}}{h+\zeta }-A\left(\frac{\partial^{2}v}{\partial x^{2}}+\frac{\partial^{2}v}{\partial y^{2}}\right)+\frac{1}{\rho }\frac{\partial p_{a}}{\partial y}-\frac{\rho_{a}}{\rho }\frac{C_{d}W_{y}\sqrt{W_{x}{}^{2}+W_{y}{}^{2}}}{h+\zeta }\right]dxdydt.$$

Analogous to the means of He et al. [26], we can obtain the adjoint equations:

(11) $$\frac{\partial \zeta_{a}}{\partial t}+u\frac{\partial \zeta_{a}}{\partial x}+v\frac{\partial \zeta_{a}}{\partial y}+\frac{ku\sqrt{u^{2}+v^{2}}u_{a}}{{\left(h+\zeta \right)}^{2}}+\frac{kv\sqrt{u^{2}+v^{2}}v_{a}}{{\left(h+\zeta \right)}^{2}}+g\frac{\partial u_{a}}{\partial x}+g\frac{\partial v_{a}}{\partial y}=K_{\zeta }\left(\zeta -\hat{\zeta }\right)\;\frac{\partial u_{a}}{\partial t}-\left[f+\frac{kuv}{\left(h+\zeta \right)\sqrt{u^{2}+v^{2}}}\right]v_{a}-u_{a}\frac{\partial u}{\partial x}-v_{a}\frac{\partial v}{\partial x}+\frac{\partial }{\partial x}\left(uu_{a}\right)\;+\frac{\partial }{\partial y}\left(vu_{a}\right)+\left(h+\zeta \right)\frac{\partial \zeta_{a}}{\partial x}+A\left(\frac{\partial^{2}u_{a}}{\partial x^{2}}+\frac{\partial^{2}u_{a}}{\partial y^{2}}\right)-\frac{k\left(2u^{2}+v^{2}\right)u_{a}}{\left(h+\zeta \right)\sqrt{u^{2}+v^{2}}}=0\;\frac{\partial v_{a}}{\partial t}-\left[f+\frac{kuv}{\left(h+\zeta \right)\sqrt{u^{2}+v^{2}}}\right]u_{a}-u_{a}\frac{\partial u}{\partial y}-v_{a}\frac{\partial v}{\partial y}+\frac{\partial }{\partial x}\left(uv_{a}\right)\;+\frac{\partial }{\partial y}\left(vv_{a}\right)+\left(h+\zeta \right)\frac{\partial \zeta_{a}}{\partial y}+A\left(\frac{\partial^{2}v_{a}}{\partial x^{2}}+\frac{\partial^{2}v_{a}}{\partial y^{2}}\right)-\frac{k\left(u^{2}+2v^{2}\right)v_{a}}{\left(h+\zeta \right)\sqrt{u^{2}+v^{2}}}=0,$$

where $\zeta_{a}$, $u_{a}$, $v_{a}$ are separately the adjoint variables of ζ, u, and v.

### 2.2. Regularization Technique

The regularization technique is usually used to solve the ill-posed inverse problem. The Tikhonov regularization technique [21] is widely used, and its major idea is presented blow.

The Tikhonov functional is structured as follows:

(12) $$f=J+J_{sta}$$

where the cost function J is given by Equation (9), $J_{sta}=\frac{\alpha }{2}{\left\|b-b_{0}\right\|}^{2}$ is the “stabilizing functional” in the Tikhonov regularization technique, α(α>0) is a regularization parameter, $b_{0}$ and b are separately the prior and optimized control variables in the model. The control variable gradient of the Tikhonov functional is:

(13) $$f_{b}=d+\alpha \left(b-b_{0}\right),$$

and the Hessian matrix of the functional is

(14) $$f_{bb}=D+\alpha I,$$

where d is the first derivative of the function J, D is the second derivative, I is the identity matrix.

It is very important for the implementation of the regularization technique to choose an appropriate parameter α, that is, calculate α to minimize the Tikhonov functional. Engl’s criterion is a feasible solution for determination of the parameter α.

Suppose that $p=b-b_{0}$, then in a neighborhood of $b_{0}$,

(15) $$f\left(p\right)\approx s\left(p\right)=J\left(b_{0}\right)+d\left(b_{0}\right)p+\frac{1}{2}p^{T}D\left(b_{0}\right)p+\frac{\alpha }{2}{\left\|p\right\|}^{2}.$$

Setting $\frac{ds}{dp}=0$, we obtain:

(16) $$\left(D_{0}+\alpha I\right)p+d_{0}=0.$$

According to Equation (16), we obtain the following formulas:

(17) $$b=b_{0}-{\left(D_{0}+\alpha I\right)}^{-1}d_{0},$$

(18) $$\frac{db}{d\alpha }=\frac{dp}{d\alpha }=-{\left(D_{0}+\alpha I\right)}^{-1}p,$$

(19) $$\frac{d^{2}b}{d\alpha^{2}}=\frac{d^{2}p}{d\alpha^{2}}=2{\left(D_{0}+\alpha I\right)}^{-1}{\left(D_{0}+\alpha I\right)}^{-1}p.$$

According to Engl’s criterion, we need to minimize the functional:

(20) $$\varphi \left(\alpha \right)=\frac{J}{\alpha },\;\alpha >0.$$

To that end, we need to solve the following equation:

(21) $$\varphi^{\prime }\left(\alpha \right)=\frac{\alpha J^{\prime }-J}{\alpha^{2}}=0,\;\alpha >0.$$

Then we obtain $\alpha J^{\prime }-J=0$. Suppose that $F\left(\alpha \right)=\alpha J^{\prime }-J$ and by applying the Newton iteration method, we can obtain:

(22) $$\alpha_{1}=\alpha_{0}-\frac{F\left(\alpha_{0}\right)}{F^{\prime }\left(\alpha_{0}\right)},$$

where α can be constantly updated. According to Equation (15), we can get:

(23) $$J^{\prime }=J_{p}\frac{dp}{d\alpha }=-d{\left(D_{0}+\alpha I\right)}^{-1}p=\alpha p^{T}{\left(D_{0}+\alpha I\right)}^{-1}p.$$

Therefore, $F\left(\alpha_{0}\right)$ and $F^{\prime }\left(\alpha_{0}\right)$ can be calculated as follows:

(24) $$F\left(\alpha \right)=\alpha^{2}p^{T}{\left(D_{0}+\alpha I\right)}^{-1}p-J,$$

(25) $$F^{\prime }\left(\alpha \right)=\alpha J^{\prime \prime }+J^{\prime }-J^{\prime }=\alpha J^{\prime \prime }=\alpha \left[p^{T}{\left(D_{0}+\alpha I\right)}^{-1}p-3\alpha p^{T}{\left(D_{0}+\alpha I\right)}^{-1}{\left(D_{0}+\alpha I\right)}^{-1}p\right].$$

As we can see from Equations (22)–(25), we need to calculate the Hessian matrix to determine the parameter α. However, the calculation of the Hessian matrix is usually very difficult. For simplicity, the regularization parameter α in Equation (12) can be regarded as a constant.

### 2.3. Numerical Experiment

**Figure 1:**
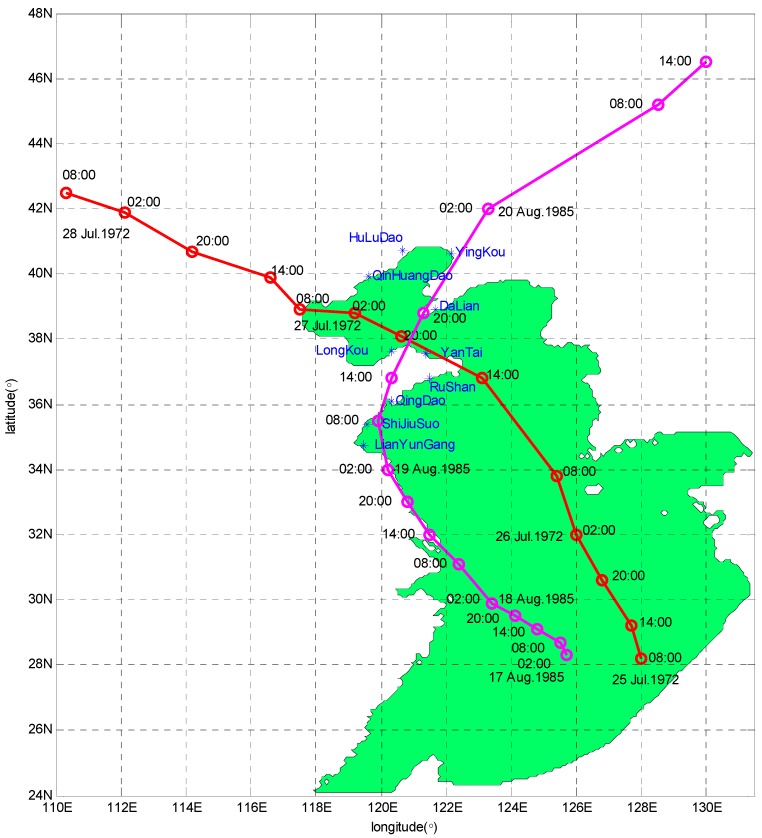
Tide stations and trajectories of Typhoons 7203 and 8509. Blue asterisks denote the locations of tide stations. Red and mauve solid lines are the trajectories of Typhoons 7203 and 8509 respectively. Red and mauve circles indicate time.

The areas studied in this paper include the Bohai Sea, Yellow Sea, and East China Sea. The specific scope of longitude is between 117.5° E and 130.5° E and the latitude is between 24.5° N and 41° N. At the initial time, sea surface level and current velocity were treated as 0 in the model. The background Cd in the area is 0.0026, which is in the range 0.002–0.004 as estimated from ocean observations [27]. The bottom drag coefficient was set to a constant (0.0016) in the whole sea area. Taiwan Strait and the first island chains were set as the open boundaries. It is supposed that no water flows into or away from the seacoast along the closed boundaries which means that the normal current component is 0. In the finite difference scheme, water elevation is at the grid center and the current speed is on the edge of grid, that is, Arakawa C is adopted. Bathymetry data was obtained from the First Institute of Oceanography, State Oceanic Administration. The horizontal resolution is 5′ × 5′. The time increment is 60 s. The model is driven by the surface stress Equation (4) and pressure Equation (8). In present study, Typhoons 7203 (from July 25 to 28, 1972) and 8509 (from August 17 to 20, 1985) were chosen for simulation. The trajectories of the two Typhoons are shown in Figure 1. Observation data from 10 tide stations was used for assimilation, and their locations are also shown in Figure 1. In order to obtain the temporally varying wind-stress drag coefficient, the typhoon process separates into a few periods, and each period lasts 6 h. Track data of typhoons used in the study are from the website “http://www.typhoon.org.cn/”.

The observation data used for assimilation were water levels, observed at tide stations during Typhoons 7203 and 8509. To implement the Tikhonov regulation technique, the Tikhonov function as Equation (7) is used. Different regularization parameters (0, 1, 10, 100, and 1000) were tested to evaluate the effect of the regularization parameter on the simulated results. Accordingly, five numerical experiments, denoted by Cases 0–4, were carried out. Then, the simulated storm surge elevations were compared with the observations. Additionally, another experiment, denoted by Case 5, was performed with the independent point method [17], for comparison to further demonstrate the efficiency of the regularization technique.

## 3. Results and Discussion

### 3.1. Comparison between Simulation and Observation of Storm Surge Levels

In this section, a sequence of comparisons between the simulations of storm surge levels and observations are carried out at several tide stations during Typhoons 7203 and 8509, and the model results are further analyzed.

**Figure 2:**
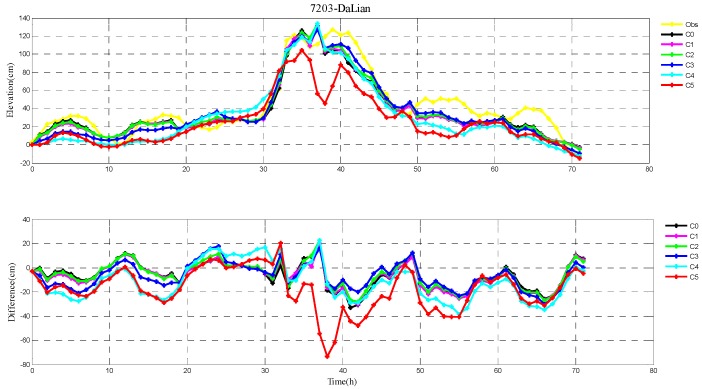
The storm surge elevations (cm) in six cases (C0, C1, C2, C3, C4, C5) and observations (yellow line) (top) and the differences between them (bottom) during Typhoon 7203 at DaLian tide station.

**Figure 3:**
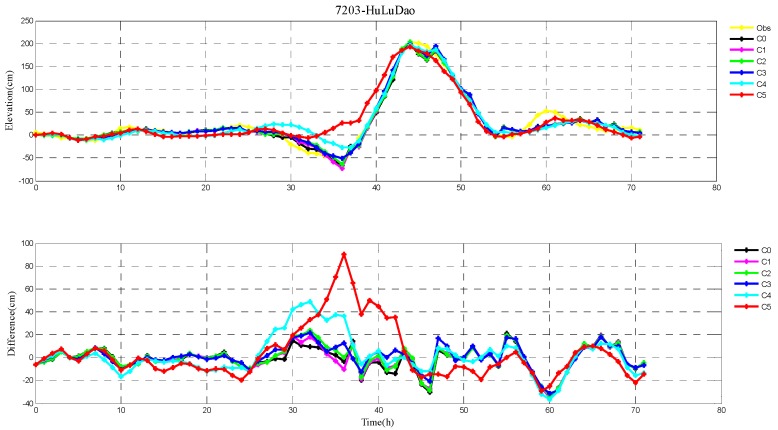
The storm surge elevations (cm) in six cases (C0, C1, C2, C3, C4, C5) and observations (yellow line) (top) and the differences between them (bottom) during Typhoon 7203 at HuLuDao tide station.

**Figure 4:**
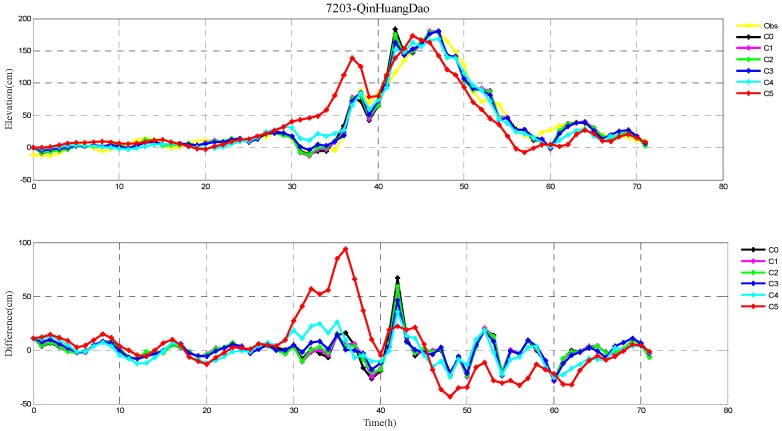
The storm surge elevations (cm) in six cases (C0, C1, C2, C3, C4, C5) and observations (yellow line) (top) and the differences between them (bottom) during Typhoon 7203 at QinHuangDao tide station.

**Figure 5:**
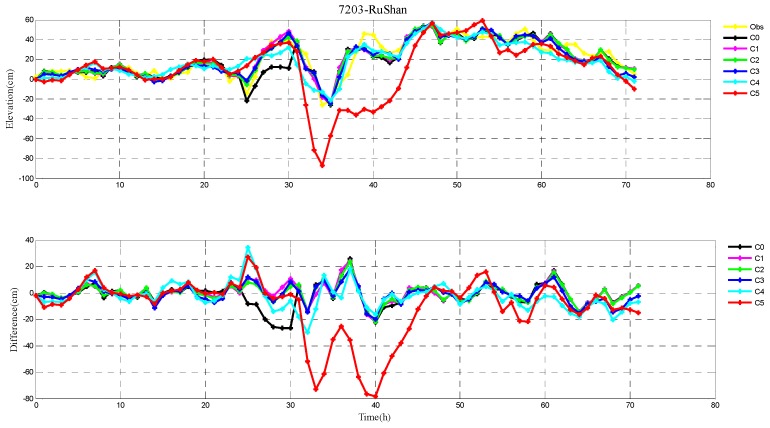
The storm surge elevations (cm) in six cases (C0, C1, C2, C3, C4, C5) and observations (yellow line) (top) and the differences between them (bottom) during Typhoon 7203 at RuShan tide station.

**Figure 6:**
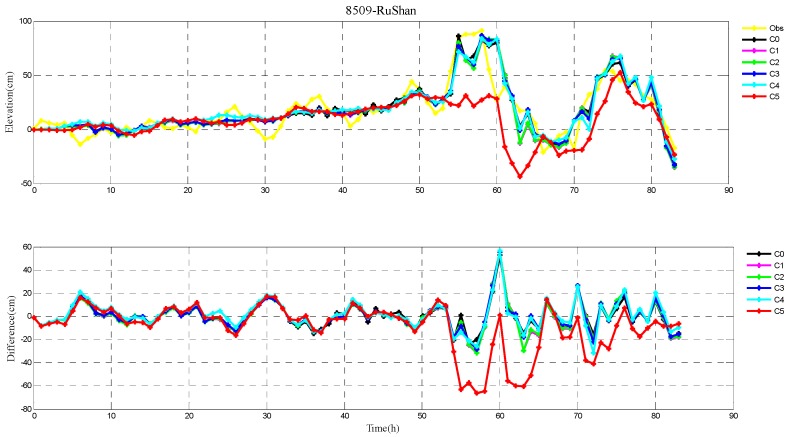
The storm surge elevations (cm) in six cases (C0, C1, C2, C3, C4, C5) and observations (yellow line) (top) and the differences between them (bottom) during Typhoon 8509 at RuShan tide station.

**Figure 7:**
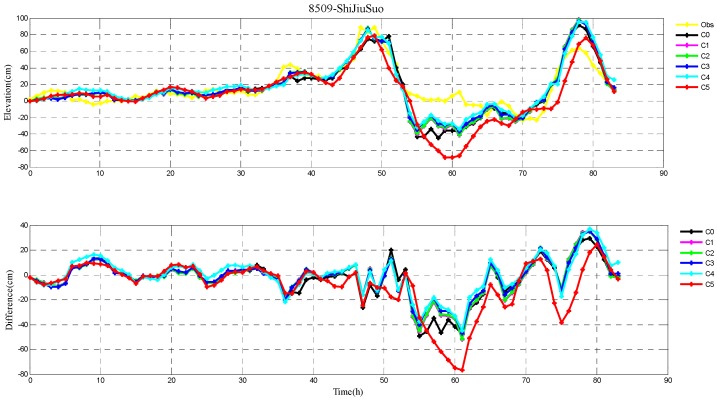
The storm surge elevations (cm) in six cases (C0, C1, C2, C3, C4, C5) and observations (yellow line) (top) and the differences between them (bottom) during Typhoon 8509 at ShiJiuSuo tide station.

**Figure 8:**
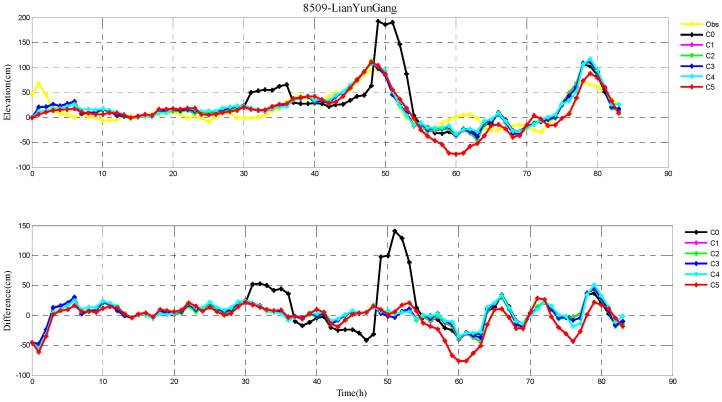
The storm surge elevations (cm) in six cases (C0, C1, C2, C3, C4, C5) and observations (yellow line) (top) and the differences between them (bottom) during Typhoon 8509 at LianYunGang tide station.

The root-mean-square (RMS) errors between simulation and observations are presented in Table 1 and Table 2 for each period during Typhoons 7203 and 8509, respectively. The RMS errors at each tide station are listed in Table 3. In addition, the comparison of the peak surge and peak time between simulation and observations at tide stations during Typhoons 7203 and 8509 are also shown in Table 4 and Table 5, respectively. Specially, the simulated and observed storm surge elevations, and their differences at DaLian, HuLuDao, QinHuangDao, and RuShan tide stations during Typhoon 7203 are plotted in Figure 2, Figure 3, Figure 4 and Figure 5, respectively. The similar results at RuShan, ShiJiuSuo, and LianYunGang tide stations during Typhoon 8509 are shown in Figure 6, Figure 7 and Figure 8, respectively.

In this study, the RMS errors between simulation and observation of storm surge levels, peak surge, and peak time during typhoons serve as criteria for determination of the best simulation. As we can see from Table 1, the mean RMS differences in C0–C5, for Typhoon 7203, are 15, 14, 14, 13, 16, and 20 cm, respectively. Particularly, the mean value in C3 is the smallest. Mean RMS differences obtained by the regularization technique are smaller than that by the independent point method. For Typhoon 8509, as shown in Table 2, the mean RMS differences in C0–C4 are respectively 23, 19, 19, 19, and 19 cm, and smaller than that in C5 (24 cm). As shown in Table 3, the mean RMS differences at all tide stations in C1–C4 are obviously smaller than those in C5 during Typhoons 7203 and 8509. From the Table 4 we can see that, at DaLian and HuLudao tide stations, both peak surge and peak time in C3 are the closest to the observation. In Case 3, the peak surge at QinHuangdao tide station is the closest to the observation and the peak time at RuShan tide station is the closest to the observation. In Table 5, the result of C3 at RuShan station is closer to the observation in terms of both the peak surge and peak time. At the LianYungang station, C4 is the best. In the C0, where the regularization technique is not employed, the simulation of the peak surge and peak time is poorer than that in C3 for Typhoon 7203, and the RMS errors are higher than those in C1–C4 for Typhoon 8509. These results demonstrate that, compared with the simulation with the independent point method, the simulation with the regularization technique is closer to observation. In addition, the results of Typhoon 7203 show that too large or too small regularization parameter may have a negative influence on performance of the method. Therefore, it is very important to choose an appropriate regularization parameter for the Tikhonov regularization technique. However, the choice of the regularization parameter has little impact on the simulation results of Typhoon 8509. These findings are consistent with the results shown in Figure 2, Figure 3, Figure 4, Figure 5, Figure 6, Figure 7 and Figure 8. 

### 3.2. Spatial Distribution of the Drag Coefficient

After comparison in terms of the mean RMS difference, peak surge, and peak time, in this section we chose the result of Case 3 as an example to investigate the spatial distribution of the inverted drag coefficient and storm surge elevation. 

**Figure 9:**
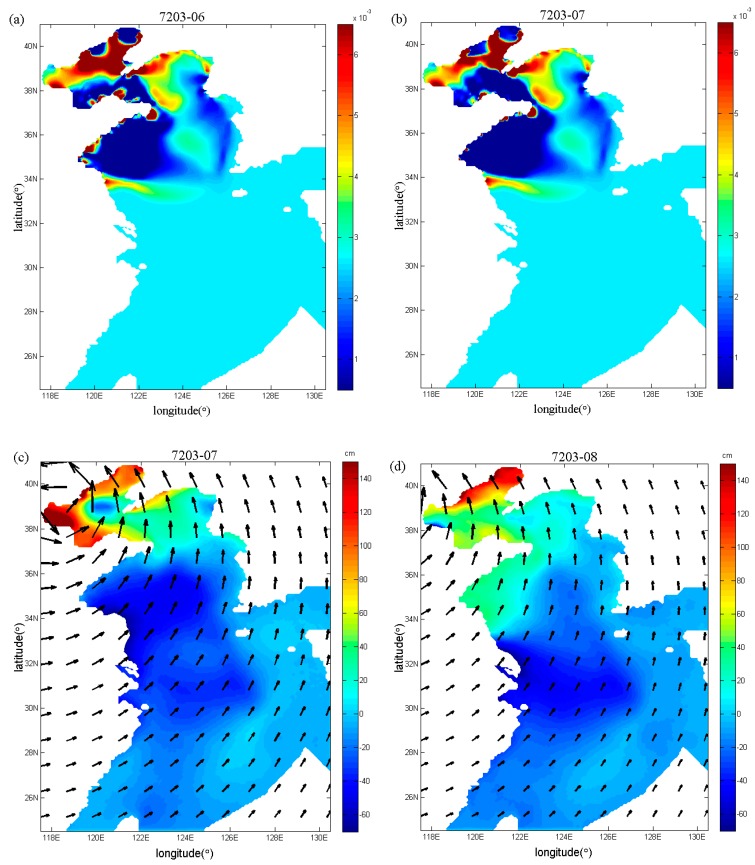
Spatial distributions (**a**,**b**) of the drag coefficient in the sixth and seventh periods and spatial distributions (**c**,**d**) of storm surge elevation and wind field in the seventh and eighth periods in Case 3 during Typhoon 7203.

**Figure 10:**
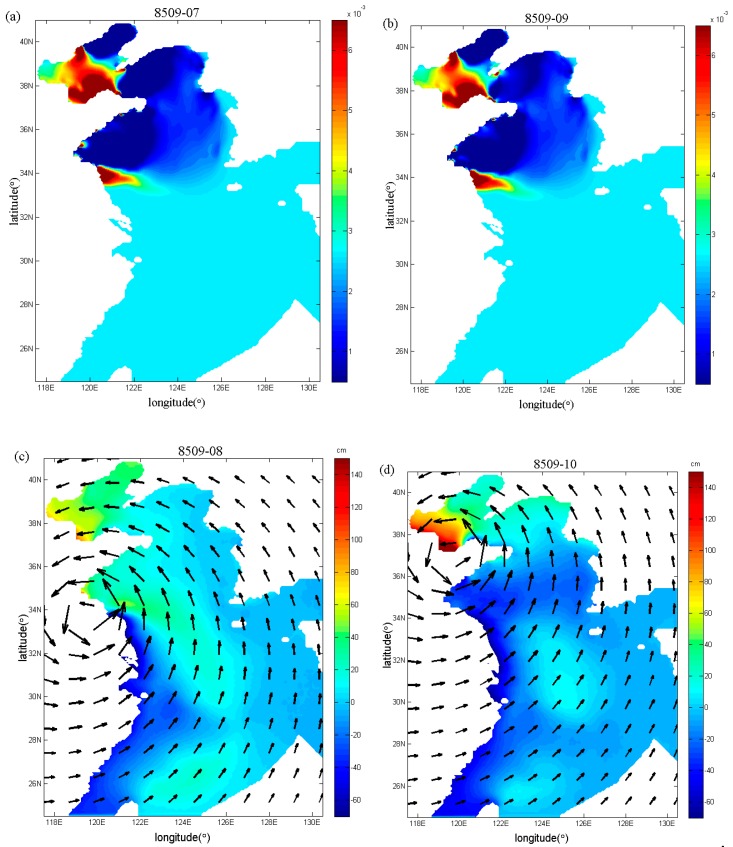
Spatial distributions (**a**,**b**) of the drag coefficient in the seventh and ninth periods and spatial distributions (**c**,**d**) of storm surge elevation and wind field in the eighth and tenth periods in Case 3 during Typhoon 8509.

**Figure 11:**
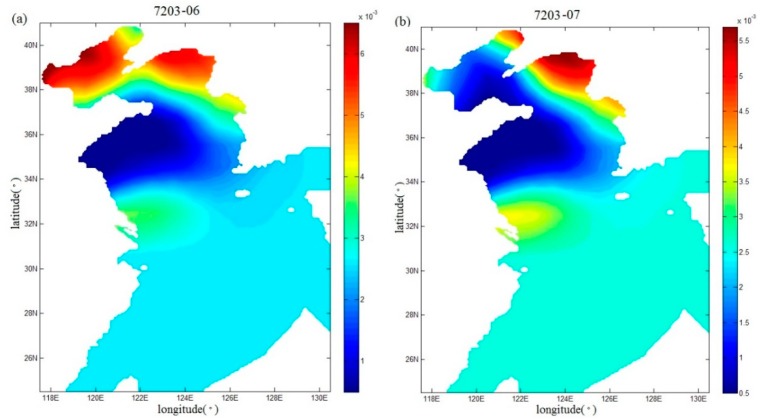
Spatial distributions (**a**,**b**) of the drag coefficient in the sixth and seventh periods in Case 5 during Typhoon 7203.

**Figure 12:**
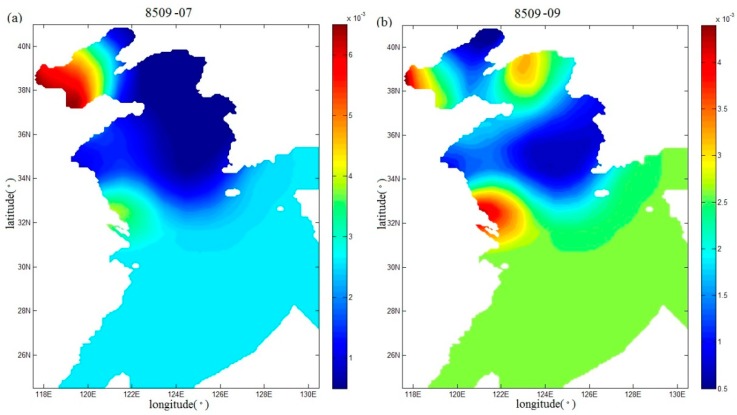
Spatial distributions (**a**,**b**) of the drag coefficient in the seventh and ninth periods in Case 5 during Typhoon 8509.

Fan et al. [17] also inverted the spatial distributions of the drag coefficient during Typhoons 7203 and 8509, but using the independent point method. In this study, in order to compare with the study of Fan et al. [17], the spatial distributions of the drag coefficient in the sixth and seventh periods (that is, the end of the sixth and seventh running periods) during Typhoon 7203 are shown in Figure 9a,b, and the spatial distributions of the drag coefficient in the seventh and ninth periods (that is, the end of the seventh and ninth running periods) during Typhoon 8509 are mapped in Figure 10a,b, respectively. From Figure 9a,b and Figure 10a,b, the drag coefficient calculated with the regularization technique show more variations than those in Figure 11a,b and Figure 12a,b. In addition, we can also find that the extrema of drag coefficients appear in the Bohai Sea and northern Yellow Sea, especially in the coastal areas, while values change slightly in the south of the Yellow Sea and nearly remains unchanged in the East China Sea. One possible interpretation for this is that the convergence and divergence of water are stronger in the shallower water areas and in the coastal areas. 

With the inverted drag coefficient in the sixth and seventh periods during Typhoon 7203, the simulated spatial distributions of storm surge elevation and wind field in the seventh and eighth periods are shown in Figure 9c,d. From Figure 9c,d, the winds mainly blow toward the land and push water toward the coast so as to raise the sea level in the Bohai Sea, but in the South Yellow Sea the negative storm surges dominate under the influence of offshore winds. Similarly, for Typhoon 8509, by using the inverted drag coefficient in the seventh and ninth periods, the simulated spatial distributions of storm surge elevation and wind field in the eighth and tenth periods are mapped in Figure 10c,d. From Figure 10c,d we can see that the negative storm surges mainly occur in the South Yellow Sea where the offshore wind plays a leading role.

## 4. Conclusions

By assimilating the observed water levels at tide stations during Typhoons 7203 and 8509 into a numerical assimilation model, the water levels in the Bohai Sea, Yellow Sea, and East China Sea were simulated. The drag coefficient in whole areas was inverted during the two Typhoons. The Tikhonov regularization technique with different regularization parameters were applied to the process of adjoint assimilation.

To study the impacts of different regularization parameters on the simulation results, five experiments, denoted by Cases 0–4, were carried out with five regularization parameters 0, 1, 10, 100, and 1000, respectively. The results showed that for Typhoon 7203, the mean RMS differences in C0–C4 were 15, 14, 14, 13, and 16 cm, respectively. Among them, the mean value in C3 was the smallest. For Typhoon 8509, the mean RMS differences in C0–C4 were respectively 23, 19, 19, 19, and 19. Meanwhile, the storm surge elevations calculated with the regularization technique were compared with those obtained with the independent point method, and the result indicates that the former one performed better.

The result of Case 3 was used for further investigation. The spatial distributions of the drag coefficients in the sixth and seventh periods during Typhoon 7203 and those in the seventh and ninth periods during Typhoon 8509 were displayed. These results demonstrate that different typhoon trajectories can lead to the different drag coefficients, and the drag coefficient has more obvious variation in the coastal waters of the Bohai Sea and northern Yellow Sea. Finally, spatial distribution of storm surge elevation and wind field during the two Typhoons were shown. These results indicate that onshore winds push water toward the coast so as to increase the water elevation in the Bohai Sea and offshore winds can excite the negative storm surge in the South Yellow Sea.

## Figures and Tables

**Table 1:** Mean square errors between simulation and observation of storm surge levels in each period during Typhoon 7203 (unit: cm).

Case	1	2	3	4	5	6	7	8	9	10	11	12	Mean
C0	11	7	5	6	11	16	29	19	22	27	20	12	15
C1	6	7	4	5	6	14	26	18	21	26	19	12	14
C2	5	7	5	5	5	16	27	18	19	25	19	12	14
C3	7	8	6	6	6	14	24	16	16	23	18	13	13
C4	10	11	10	9	11	20	22	19	20	23	19	15	16
C5	10	11	10	9	8	35	45	32	24	21	22	14	20

**Table 2:** Mean square errors between simulation and observation of storm surge levels in each period during Typhoon 8509 (unit: cm).

Case	1	2	3	4	5	6	7	8	9	10	11	12	13	14	Mean
C0	11	8	4	7	10	20	11	15	39	23	31	74	32	37	23
C1	11	7	4	7	10	9	7	10	7	21	32	66	36	43	19
C2	11	7	4	7	10	9	7	10	7	21	31	66	35	42	19
C3	11	8	4	7	10	10	8	9	7	19	29	67	34	40	19
C4	13	11	6	8	12	11	10	8	8	18	27	69	32	39	19
C5	13	7	5	9	10	9	7	11	11	35	49	101	38	30	24

**Table 3:** Mean square errors between simulation and observation of storm surge levels at tide stations during Typhoons 7203 and 8509 (unit: cm).

Tide Stations	7203						8509					
	C0	C1	C2	C3	C4	C5	C0	C1	C2	C3	C4	C5
DaLian	13	13	12	13	19	25	56	50	50	50	52	73
YingKou	13	11	11	12	14	19	*	*	*	*	*	*
HuLuDao	10	10	11	10	16	23	*	*	*	*	*	*
QinHuangDao	11	10	10	9	11	27	27	31	31	29	27	27
LongKou	19	17	18	16	19	23	*	*	*	*	*	*
YanTai	26	26	26	22	26	27	22	24	23	22	21	23
RuShan	9	7	7	7	10	25	10	11	11	11	11	19
QingDao	16	15	15	15	14	19	14	14	13	13	12	20
ShiJiuSuo	18	15	15	13	12	19	16	15	14	14	14	24
LianYunGang	26	23	23	20	17	25	34	16	16	16	17	25
Mean	16	15	15	14	16	23	26	23	23	22	22	30

* denote the absence of observations.

**Table 4:** The peak surge and peak time between simulation and observation of storm surge levels at four tide stations during Typhoon 7203.

Typhoon	7203							
Tide Stations	DaLian		HuLuDao		QinHuangDao		RuShan	
	Peak surge (cm)	Peak time (h)	Peak surge (cm)	Peak time (h)	Peak surge (cm)	Peak time (h)	Peak surge (cm)	Peak time (h)
C0	135	35.6	211	43.6	185	42	55	46.6
C1	136	36.3	213	43.7	182	45.9	54	46.5
C2	136	36.3	212	43.7	181	45.8	56	46.5
C3	134	36.3	204	43.7	181	46.9	57	46.9
C4	137	36.3	199	43.8	172	43.6	58	47.5
C5	106	35.3	193	43.9	173	43.0	62	52.7
Observation	127	39	204	44	181	46	53	47

**Table 5:** The peak surge and peak time between simulation and observation of storm surge levels at three tide stations during Typhoon 8509.

Typhoon	8509					
Tide Stations	RuShan		ShiJiusuo		LianYungang	
	Peak surge (cm)	Peak time (h)	Peak surge (cm)	Peak time (h)	Peak surge (cm)	Peak time (h)
C0	91	55.2	92	50.6	196	49
C1	84	58.3	88	48.1	113	48
C2	85	58.3	88	48.1	113	48
C3	88	58.2	87	48.1	111	48
C4	84	58	86	48.1	109	48
C5	53	75	79	48.5	114	48.3
Observation	92	58	89	49	95	48
